# The Role of Soft Robotic Micromachines in the Future of Medical Devices and Personalized Medicine

**DOI:** 10.3390/mi13010028

**Published:** 2021-12-26

**Authors:** Lourdes Garcia, Genevieve Kerns, Kaitlin O’Reilley, Omolola Okesanjo, Jacob Lozano, Jairaj Narendran, Conor Broeking, Xiaoxiao Ma, Hannah Thompson, Preston Njapa Njeuha, Drashti Sikligar, Reed Brockstein, Holly M. Golecki

**Affiliations:** Department of Bioengineering, University of Illinois Urbana-Champaign, Urbana, IL 61801, USA; lourdes3@illinois.edu (L.G.); gmkerns2@illinois.edu (G.K.); kro5@illinois.edu (K.O.); omolola2@illinois.edu (O.O.); jlozan20@illinois.edu (J.L.); jnaren2@illinois.edu (J.N.); conorab2@illinois.edu (C.B.); xm15@illinois.edu (X.M.); hst2@illinois.edu (H.T.); pn4@illinois.edu (P.N.N.); dsikli2@illinois.edu (D.S.); reedlb2@illinois.edu (R.B.)

**Keywords:** soft robotics, biomaterials, medical devices, wearable technologies

## Abstract

Developments in medical device design result in advances in wearable technologies, minimally invasive surgical techniques, and patient-specific approaches to medicine. In this review, we analyze the trajectory of biomedical and engineering approaches to soft robotics for healthcare applications. We review current literature across spatial scales and biocompatibility, focusing on engineering done at the biotic-abiotic interface. From traditional techniques for robot design to advances in tunable material chemistry, we look broadly at the field for opportunities to advance healthcare solutions in the future. We present an extracellular matrix-based robotic actuator and propose how biomaterials and proteins may influence the future of medical device design.

## 1. Introduction

**Figure 1:**
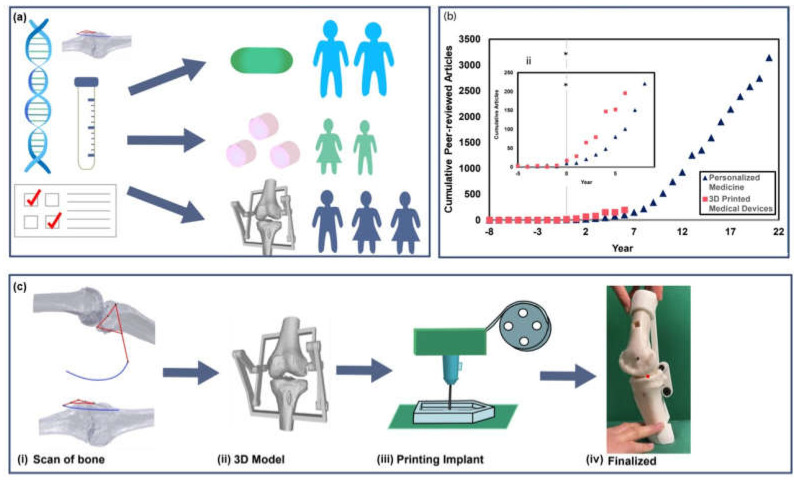
Future outlook for personalized medical treatments. (**a**) Schematic illustration of the application of genetic testing, medical imaging, and diagnostics for creating personalized treatments for a range of patient cases. (**b**) Analysis of cumulative publications on “personalized medicine” (blue) and “3D printed medical devices” (red) compared against a benchmark (* year 0) of 10 paper per year in each field. Inset shows exponential growth in both fields in years 0 through 7 from benchmark. Analysis performed on data collected from Web of Science. (**c**) Example of 3D printed implant models created from CT scans, showing progression from imaging to a patient specific model, followed by printing and an image of a finalized implant. (Images c(i), (ii), (iv) adapted from Mercader et al. 2021 [5] http://creativecommons.org/licenses/by/4.0/ (accessed on 1 December 2021)).

Personalized medicine, tailored treatments for patient-specific physiology, genetic makeup, and health history (Figure 1a), have induced a shift towards preventative care methods [1]. The evolution of medical devices, therapeutics, and surgical tools enables practitioners to advance personalized medicine approaches including incorporating low modulus materials into medical devices. As a result, continuous and real-time health monitoring, point-of-care testing technologies, and patient-specific 3D printed devices have come to the forefront of biomedical research. Figure 1b shows the cumulative articles published on “personalized medicine” over the past 22 years since the field reached a publication benchmark of 10 papers per year (* at year 0 in Figure 1b inset). We compared “personalized medicine” to a more specific area, “3D printed medical devices”, a field in which a patient’s anatomy can be scanned and replicated or altered in an additive manufactured model. While “3D printed medical devices” is a comparatively new area of research, when the fields are compared against the benchmark (*), both fields experienced exponential growth in the first five years post benchmark. This analysis suggests that beyond personalized therapeutic treatments, new manufacturing methods are critical for the next generation of personalized medicine. The progression of medical diagnostics and treatment methods toward personalized medicine and point-of-care diagnostics shows promise in increasing affordability, safety, and clinical efficacy of medical care [2,3]. With these developments, arises the need for new tools and devices to provide specialized care. Advances in biomedical imaging and additive manufacturing across a range of material moduli have enabled production of biomimetic and personalized implants, such as a 3D printed knee for total knee arthroplasty (Figure 1c) [4]. In this paper, we present a review of fabrication techniques and applications of soft robotic devices to address these biomedical challenges.

**Figure 2:**
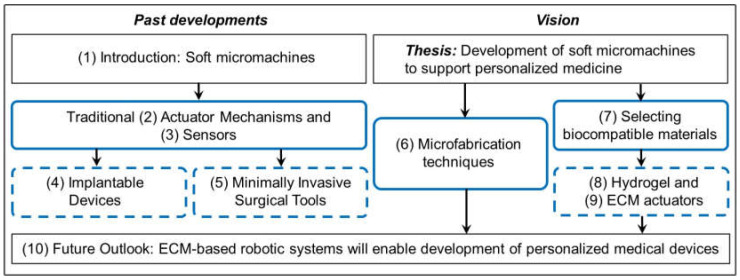
Overview of this review including sections and thesis. Section number in (), solid black line outlines themes and thesis, solid blue lines outline fabrication techniques, and dashed blue line outlines applications.

The field of soft robotics integrates low modulus materials into robotic systems to increase safety in human-robot interactions. These compliant devices have numerous biomedical applications due to their biocompatibility, range of motion, material compliance, and tunability [5,6]. With the integration of personalized medicine into the traditional healthcare system, we will explore how soft robotics may provide opportunities for the development of patient-tailored medical devices and increased comfort and biocompatibility for continuous health monitoring devices. Figure 2 details the organization of this review, examining the field of soft robotics and its application in the future of personalized medicine across multiple spatial scales of the body.

This review begins by examining the evolution of the field of soft robotic systems and methods of manufacturing, then highlights applications of soft robotics as both implantable devices, tools for minimally invasive surgery, and wearable technologies. These applications allow for customized approaches to monitoring and treatment of chronic illness, serious injuries, cardiovascular diseases, and cancers. We show that wearable sensors, such as those used for chronic disease management, have increased the accessibility and affordability of point-of-care testing allowing for personalized care and subsequently increased patient compliance [3]. Next, we motivate a vision for the role soft robotics can play in the future of personalized medicine by combining miniaturization of robotic components at the micro and nanoscale as well as incorporating biopolymer and protein-based systems into medical devices. This combination rests at the intersection of robotic and personalized medical techniques as it incorporates the benefits of autologous treatment methods to improve patient response and reduce undesirable outcomes of synthetic implants, such as uncontrolled immune response. We believe the use of microscale robotics and autologous biomaterials in creating dynamic, functional devices, and tools holds promise for advancing personalized medicine.

## 2. History of Actuator Development and Applications in Wearable Technologies

Wearable devices such as gloves and exosuits represent some of the most well-developed applications of soft robotics in healthcare [2,3,4,5]. Wearable devices used for assistive and rehabilitative purposes restore limb and joint function. Advances in materials increases comfort and usability of such devices [6,7,8,9,10]. Fundamentals from the fabrication techniques used to build suit and glove devices may provide inspiration and inform design of microscale counterparts. The following subsections highlight a historical view of actuator mechanisms employed in soft wearable devices to date.

### 2.1. McKibbens Actuators

**Figure 3:**
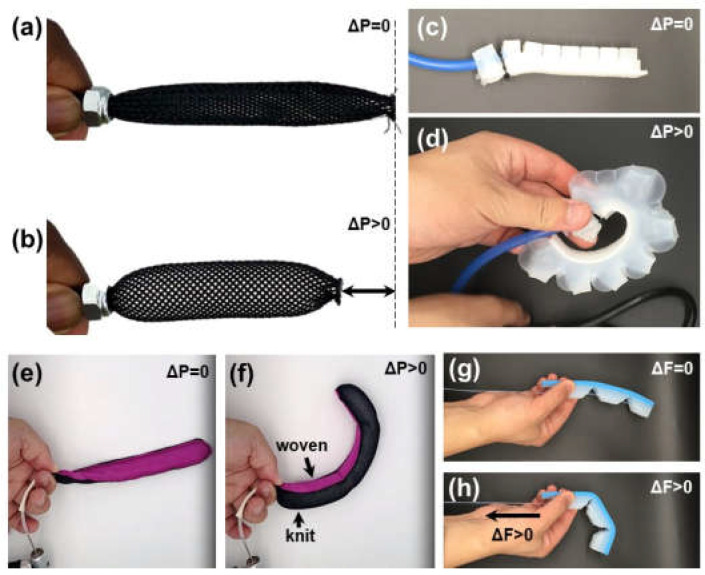
Examples of soft robotic actuators. (**a**) McKibbens style actuator relaxed, and (**b**) contracted by air powered shortening. (**c**) FEA style actuator built according to the protocol reported in Greer et al. [33] and (**d**) curled with air pressure. (**e**) Textile actuators curl based on textile weave [33]. (**f**) The restrictive weave of the pink fabric causes the actuator to curl toward woven fabric while the knit is extensible and allows for expansion. (**g**) Tendon actuated finger (**h**) curls toward phalanges due to compliant hinges in the design.

One of the earliest soft actuators used for biomedical applications was developed in 1958 by Joseph Laws McKibben to pneumatically activate forearm orthotics [11]. Named after its creator, McKibben actuators consist of a rubber bladder enclosed in a helically wound mesh [12] (Figure 3a). The pneumatic inflation of the bladder coupled with the longitudinal stiffness of the fibers produces actuator shortening and tensile forces (Figure 3b). Mechanical programming in McKibben actuators set the blueprint for pneumatic artificial muscles (PAMs) that are still used today [13]. PAM structures allow for unidirectional, contractile shortening, functioning similarly to human muscle fibers in vivo. Recently, an elbow and forearm rehabilitation device used two pairs of two antagonistically oriented PAMs to enable two degrees of freedom: flexion and extension of the elbow and pronation and supination of the forearm [14,15,16]. Shiota et al. used PAMs to create an assistive device for thumb rehabilitation [17]. The device was able to assist in the thumb’s abduction, adduction, and opposition movements. Recent efforts include fabrication of coupled microscale PAMs [17] and their application in vivo to assist cardiac contractile function [18]. Though now characterized under the ever-growing umbrella of pneumatic actuators, McKibben actuators were a key first step in the development of soft actuator mechanisms for wearable, medical applications.

### 2.2. Silicone Pneumatic Actuators

**Figure 4:**
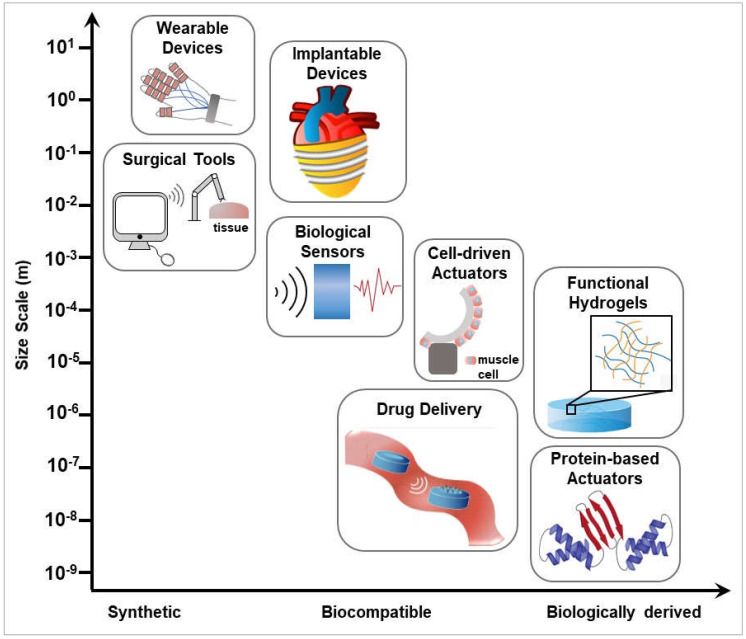
Summary of materials and techniques used in the development of soft robots and medical devices. Techniques are organized by size scale (*y*-axis) and material chemistry (*x*-axis) from synthetically derived to biologically sourced materials.

Later, flexible pneumatic microactuators molded from elastomer materials were pioneered by Koichi Suzumori in his 1992 work [19,20,21]. This design consists of a fiber-reinforced rubber cylinder with three independently pressurized chambers to control bending direction. The principles behind this work have more recently evolved into the branch of fluidic elastomer actuators (FEAs) commonly used today [21]. FEA designs span actuator geometries [22,23] built from a variety of low modulus, non-linear elastomeric polymers [24,25,26,27]. Almost two decades after Suzumori’s work, came the development of the PneuNet actuator [28]. Named for characteristic pneumatic networks, these composite elastomers of connected chambers (Figure 3c) actuate in a balloon-like fashion (Figure 3d). The silicone, extensible side of the actuator allows for expansion upon inflation and a fabric reinforced, inextensible side constrains motion, resulting in a highly non-linear conformation change upon actuation, similar to finger flexion. Extensible and inextensible constraints can be created by embedded or sleeved fibers, as seen in Figure 3e,f in the example of textile actuators [29]. Diemel et al. created “PneuFlex” actuators which are based on the same principles as PneuNets [30,31] but reinforced with helically wound polymer fibers to increase durability. This design created a robust gripper capable of manipulating objects weighing up to half a kilogram. Today FEAs are built to mimic biomimetic organisms [21,22] and assembled in soft wearable systems [10,30,31,32]. The finger-like movement of FEAs is leveraged to create soft gloves for assistive finger movement and rehabilitation (Figure 4) to create a biomimetic glove [31]. Elastomer-based FEAs are scalable for microscale applications using techniques such as soft lithography [33,34]. The lower limit of the device size is only limited by fluid power supply.

### 2.3. Tendon-Based Actuation

Tendon-driven mechanisms are often used in conjunction with more passive soft materials in actuators for rehabilitative devices. The tendon-like cables are most commonly powered by servo motors and link multiple segments of shaped soft materials to create contractile movements [35]. Figure 3g,h shows silicone cable-based actuators in relaxation and flexion. Similar cable-driven mechanisms are integrated in upper-body exosuits to actuate multiple arm or finger joints individually [36]. Awad et al. developed a wearable soft exosuit to combat abnormal stroke-induced gait [7]. The exosuit uses Bowden cable-driven actuators to coordinate dorsiflexion and plantar flexion to allow greater ground clearance and forward propulsion to decrease the metabolic expense of abnormal gait. Recently, a lightweight, backpack-like exosuit, Auxilio, was developed as a rehabilitative device to support the movement of multiple joints [8]. Auxilio exerts cable-driven force via DC motors to promote shoulder flexion and abduction as well as elbow flexion. Limited by the size of wires and motors, cable-based actuation systems are extremely scalable for both macro and micro applications.

### 2.4. Jamming Structures: Stiffening as a Mechanism for Shape Change

Material jamming is characterized by an outer membrane containing a filler material that collapses in a controlled manner under applied vacuum. Upon contraction the density of the enclosed material increases, increasing the rigidity of the jamming structure [37]. Particle or granular jamming, which is characterized by small granules, such as coffee grounds or sand, inside a nonporous elastomeric bag is capable of conforming to irregularly shaped objects [37] and operating in space-constrained environments [38]. Laminar or planar jamming is achieved by compressing sheets of material inside an elastic container [39]. Hauser et al. developed JammJoint, a soft wearable robot that uses a granular jamming to vary stiffness [40] to support elbows or knees during extenuating movements. A linkage-based layer jamming mechanism was developed by Choi et al. to give support to the spine, wrist, forearm, or elbow as a mechanism for injury prevention and rehabilitation [41]. Although jamming structures require tubing connected to a vacuum, often critiqued for being bulky, advances in pneumatic technology offer solutions to bring the pneumatics on board as devices including particle jamming are developed [42].

### 2.5. Dielectic Elastomer Actuators

Dielectric elastomer actuators (DEAs) leverage a pair of parallel electrodes sandwiching a dielectric to produce bending modes. When a voltage difference is applied across the electrodes, elastomers expand in the same plane as the electrode plates due to electrostatic pressure [43]. When designing such systems, dielectric materials with high dielectric constant, low elastic modulus, and low viscosity such as acrylics, silicones, and polyurethanes are ideal candidate materials. For electrodes, high compliance and conductivity are essential for which graphite, silver nanoparticles, and carbon black are common choices [44]. DEAs have been widely researched for their applications in grippers due to their tunable actuation configurations, lightweight nature, and high energy density [43,44]. They have also been proposed as an artificial muscle due to large deformation and responsiveness [45]. Choi et al. utilized DEAs as artificial muscles to allow walking-like locomotion in their biomimetic hexapod [46]. Menon et al. developed a DEA controlled, electrically tunable, compression bandage capable of exerting pressure on the calf muscles to treat inadequate blood circulation in lower extremities [47]. The simplicity of DEA construction means they can be reproduced on a microscale. In fact, recent research has yielded the development of miniature DEAs with dimensions on the hundreds of micrometer scale opening the door to future microscale applications of such devices [48].

### 2.6. Shape Memory Alloys

Shape memory alloys (SMAs) have long been used as a contractile mechanism in robotic actuators [49]. Heating an SMA takes it to its extensible austenite state while cooling will transform it to its rigid, folded martensite state [50]. SMAs are an alternative to traditional pneumatic actuators due to their ease of scalability, light-weight components, and unchanging mechanical properties in various fluid environments [51]. Their arrangement into coiled springs, meandering and custom geometries allow for varying deformations and applied forces [52]. SMAs have been used to create biomimetic structures such as inchworms [52,53], rays [54], and jellyfish [52]. SMAs are intrinsically compliant. Kim et al. developed SMA-based smart sock or brace for plantar flexion ankle assistance [55]. SMA-based fabric muscles have been used to give support to the spine and shoulders [56]. SMA wires inside Bowden cables have allowed for the development of various arm exoskeletons, including active actuation for wrist extension and passive wrist flexion [50]. Recently, SMA torsion springs were fabricated using treated nitinol wire for image guided surgical procedures [57]. Miniaturization of SMA muscles is supported by recent 200 μm diameter Cu-based single crystals capable of the martensitic transformation characteristic of SMA behavior [58]. This recent work demonstrates the application of miniaturized SMA actuators in microscale applications.

### 2.7. Magnetic Actuation

Magnetic actuation is a relatively new technique in soft robotics. Therefore, there is no one widely accepted procedure that wholly encompasses this technique. Instead, magnetic actuation serves as an umbrella term for methods that use magnetic materials in the form of thin metal films, conductive particles in a flexible substrate, and liquid metals [59]. Generally, magnetic actuation is characterized by the placement of the magnetic material, with or without applied current, in an external magnetic field resulting in magnetization and a subsequent conformation change [60]. Rui et al. used the widely used liquid metal gallium-indium alloy in a coil configuration sandwiched between PDMS layers to create a soft jellyfish, soft fishtail, and a soft manipulator on the centimeter scale [61]. Current through the coil in the presence of an external magnet, stretches the coil and applies uniaxial bending. This technology has also been used to create other biomimetic robots such as snakes and tadpoles [61,62]. Diller et al. [63] designed a gripper using a permanent magnet and a switchable ferrite magnet which act like jaws which can be driven open and close to create a grip and release mechanism in the presence of an external magnetic field. Liquid metals have been used in many microscale sensor-based applications as well as mechanical actuation and their low toxicity makes them a good candidate for wearable devices and other soft robots with human interaction [59,63].

With the wide variety of actuator mechanisms present, there are multiple effective ways to enable or replicate healthy or enhanced physiological movements. The actuation mechanisms described here are primarily demonstrated in externally worn devices. However, recent advances have made scaling down these common structures a possibility for implantable solutions. Soft robotic actuators implemented in wearable technologies inside or outside the body, rely on patient feedback through sensors. Section 3 presents a brief overview of some examples of sensors incorporated in wearable devices.

## 3. Soft Sensors

To integrate robotic devices into the human body it is necessary for devices to sense their environment and take cues from patient physiology (Figure 4). Soft and flexible sensors offer the ability to integrate with the wearer while providing feedback to human-in-the-loop devices [64]. For example, a flexible, resistive cardiac sensor allows for heart rate monitoring during movement and collects electrical data on cardiac function because flexible electronics allow for skin contact [65]. A similar topical skin patch made from graphene allows real-time measurement of a pulse waveform [66]. Another biosensor performs immunoassays by identifying protein markers from blood samples. The sample enters the biosensor membrane and is analyzed in multiple assays to send its data to a patient’s smartphone [67]. Yet another biosensor modifies cotton threads to monitor diabetes and kidney metabolics. The threads absorb and sense glucose and urea levels through human sweat, providing a less invasive sensor than the traditionally used blood test [68]. These sensors represent development of wearable skin-based sensors to collect and transmit data on demand, thus increasing the accessibility and portability of such diagnostics. Advances in biodegradable electronics will also allow sensor degradation after use to eliminate the need for additional procedures to remove epidermal implants. This technology increases accessibility of healthcare, by making sensors portable and durable [69].

A wide range of techniques have been proposed to enable mass production of these sensors, including tattooing, 3D printing, and inkjet printing. These methods are relatively inexpensive, allowing for device cost reduction and increased accessibility [70]. One method, outlined by Hughes et al., creates a soft sensor that embeds conductive particles inside of a silicone matrix that can be manufactured rapidly. One example is an insole that measures pressure, while another is used to measure heart and breathing rate as well as ambulation [71]. Biological cells serve as inspiration for other biosensors: collections of sensors are assembled analogous to cells assembled in tissues. These cells report their deformation in response to haptic stimuli. Light is diffused and the amount reflected contributes to the information that the sensor gives about the haptic stimulus [72]. Bioinspired sensors will be critical to assembling future soft robotic devices for both implantable devices and scaled down tools for minimally invasive surgery. The field of soft sensors and electronics are rapidly expanding. For more extensive reviews on soft sensors see references [73,74,75].

## 4. Implantable Soft Robots

Composed of low modulus materials, soft robots are ideal for implantable devices as their physical properties can be tuned to match those of biological tissues. Soft robots are capable of coordinated and highly dynamic movements lending themselves to cardiac applications. Compliance matching is important for cardiac support as the heart is a highly dynamic organ (Figure 4) [76]. In one study, McKibben actuators were wrapped around the heart ventricles, which allowed for synergistic contraction [76]. An elastic sleeve encases the actuator, which stores energy as it is stretched when blood flows in reducing the pressure needed to contract the device. Further, coupling rings are attached on either side to contribute to the total mechanical energy applied to the heart [18]. Actuation period, rate and mechanical coupling can improve cardiac function: cardiac output is increased without loss to blood volume output [76]. A second robotic sleeve can support cardiac contraction by modeling fetal heart development—first as a sheet, and then wrapping around both vertically and laterally to create the recognizable cardiac anatomy [18]. Made of silicone, the material’s elasticity gives the sleeve mechanical properties that closely mimic cardiac tissue throughout the cardiac cycle. In vivo, the sleeve was able to restore blood output and assist in restoring cardiac function in hearts with induced acute heart failure. A third implantable device targets secondary mitral valve regurgitation when blood regurgitated through the valves causes reduced cardiac output [77]. This device is surgically implanted into the interventricular septum. Soft actuators are connected through a shaft and sit on the outside of the left ventricle. Actuator pressurization contracts the muscle, inducing ventricular contraction and changes to the shape of the ventricular muscle and mitral valve. When tested on porcine hearts, the device supported mitral valve function and left ventricle contraction [77]. These implantable soft robotic devices emphasize the use of both biomimicry and integration with existing structures to create more long-term solutions to heart failure.

The mechanical energy produced by the heart can also be utilized to power devices. One implantable device uses a triboelectric nanogenerator, which can store biomechanical energy produced in vivo. This energy can then power a device that can monitor cardiac output, effectively self- powering its own monitoring system. This device relays information wirelessly, allowing data monitoring on portable devices including smartphones [78]. This device can monitor and actuate muscle cell precursors, or myoblasts, electrically to control their differentiation and proliferation. A self-power function is achieved by combining cardiomyocytes with piezoelectric nanofibers to harness mechanical energy from the human body to electrically power devices. The distribution of cardiomyocytes aligns to increase the contraction force which deforms the microstructures of a piezoelectric material, polyvinylidene difluoride (PVDF), to generate electricity. The efficiency is determined by cellular alignment and PVDF microstructure. The design provides self-powered robots for use in vivo for potential long-term human health monitoring [79]. The future of medical treatments may include implanting this sheet onto muscle tissue to monitor and stimulate a targeted site in vivo. As soft robotic systems often mimic muscle structure, their applications in vivo are only set to expand as questions of compliance matching, surface chemistry, and biocompatibility are answered.

## 5. Compliant Minimally Invasive Surgical Tools

Minimally invasive surgery has surged in popularity due to a lower risk of patient trauma in comparison to traditional procedures. Developments in microscale technologies (Figure 4) have allowed for improved compatibility and patient safety but have also left surgeons with limited haptic feedback and a lack of realistic feel during surgery [80]. Their implementation often results in equipment lacking the force generation and maneuverability desired by surgeons. Therefore, there has been an increased focus on developing solutions to fine-tune device actuation and offer controlled-stiffness end effectors. Although current prototypes are unable to meet the dynamic abilities optimal for medical use, advancements suggest exciting capabilities for the next generation of devices [80].

### 5.1. Endoscopic Imaging

Endoscopes are equipped with fiber optic cables to illuminate and visualize tissues. Modern endoscopes average a few millimeters in diameter and are composed of polymer or glass optical fibers. Diodato et al. investigated soft manipulators equipped with camera modules as possible replacements for the traditional flexible endoscope [81]. The manipulators are equipped with three longitudinal fluidic chambers, reminiscent of Suzumori’s design [19] that allow for omnidirectional bending and allow precise angular control compared to traditional clinical endoscopes. Physical tension generated by cables forces the endoscopic head to bend. By increasing angular flexibility, additional views of the target area are available to surgeons during operation while maintaining use of robotic operating systems for teloperational control.

Diagnostics and imaging can also be done by magnetically actuated, untethered rigid endoscopic capsules [82]. These devices, just larger than typical medicinal tablets, have been improved upon with the use of compliant elastomer materials [83] increasing compatibility for use in the human body. The soft material reduces injury risk due to tissue stress, which is common with traditional rigid capsules [84]. Finally, the biocompatibility of materials used allows for excretion through passive peristalsis. Together the device allows imaging deep within organ systems without compromising procedure safety. Untethered millimeter-sized robots allow for the exploration of certain previously inaccessible biological areas inside the human body. Data collected were dependent on the device orientation in the body. The use of autonomous untethered robots allows for the expansion of active monitoring by adding mobility to the device [85]. The imaging capabilities can be combined with device tracking techniques such as fluoroscopy, radio wave transmission, or magnetic localization. Through this, the development of real time 3D visual reconstruction of organs is possible [86]. The predominant use of such devices is in post-procedure settings but future developments could allow for a wider array of applications such as more accurate diseases diagnosis and surgical risk mitigation. Similar technologies have the potential to make surgeries safer and allow for faster patient recovery.

### 5.2. Tissue Manipulators

Growth of minimally invasive surgical techniques require scaled-down instrument design. However, the reduction in size has a noticeable impact on the surgeons’ dexterity [87]. A possible solution for overcoming scaling issues are microelectromechanical systems (MEMS) pop-up devices [88]. While initially compact, the small size of the device allows for it to be delivered to the necessary location with a traditional endoscope. Once the device arrives at the desired location, it activates its pop-up mechanism. This mechanism is initiated through Sarrus linkages incorporated with pressurized fluidic micro actuators. The device applies the required tension of typical endoscopic procedures to the targeted tissue and allows for greater manipulation and control by the surgeon. Alterations to the end effector may be made to complete an array of tasks.

One device in development, known as CYCLOPS, can support existing platforms used in gastrointestinal procedures and ease the difficulty of minimally invasive surgeries [89]. The inflatable structure folds around existing flexible endoscopes and can be activated to increase radial stiffness at specific locations. The two primary components of the instrument are a cable sheath and a multilayered support structure. Inflation of one compartment of the support structure leads to volume expansion while the second compartment stiffens and exerts force on surrounding tissues. The sheath then allows for the passage of force-transmission cables that controls end-effector endoscopic tools. An increased area of maneuverability accompanied by the capability of automatic application of forces allows surgeons to regain dexterity lost in some minimally invasive surgeries.

Robotic-assisted and minimally invasive surgical techniques require extensive training. Many new devices in this field are inherently difficult to operate and thus warrant additional training to reduced surgical risk. A proposed solution is a device that in its collapsed form, is attached to an endoscope by a retractable sheath deploying the device [90]. When deployed, pneumatic actuators facilitate vacuum gripping of tissue samples. Although prototypes were constructed using a single vacuum, an array of grippers can be utilized for excising large portions of tissue.

With the shift toward using low modulus materials in the field of surgical robotics, there is a call to maintain some properties of traditionally used equipment. In many circumstances, the decreased stiffness presents challenges in the surgical setting. Nature can serve as an inspiration to solve this challenge. Observations of octopus muscle structures have led to innovative concepts for soft manipulators to incorporate similar antagonistic stiffening mechanisms [36]. A two-part system combining pneumatic and tendon actuators creates an antagonistic pair. Pneumatic actuators bend and deform the manipulator while the tensile strength of the embedded tendon works to stiffen the soft material. Variable stiffness allows for smooth maneuverability in the body while also switching to apply force when required.

The development of minimally invasive techniques have increased the efficiency of traditional tissue biopsies, such as the design of a wireless soft endoscopic capsules with magnetically actuated fine-needle biopsy capabilities [91]. In this case, the tethered capsule is equipped with an endoscope camera, a magnet used for alignment in the stomach, and a needle for collection of biopsy samples. The needle is controlled through a Sarrus-linkage which converts circular motion into linear motion to minimize the size of the device. Once the procedure has been completed, the robot can be removed from the body via the connected tether to prevent contamination and the possibility of trauma in the intestine.

Developments in tissue biopsy technology have also given rise to completely soft devices in the form of self-folding microgrippers [92]. The microgrippers are created from a hydrogel and a biodegradable polymer. These two materials offer reactionary responses to changes in the biological environment and alterations to the mechanical structure, respectively. Addition of magnetic nanoparticles in the grippers also allows for changes in location and targeted delivery using magnetic probes. The star-shaped microgripper’s arms close their grasp when exposed to the specified stimuli, entrapping a sample of cells. Due to their small size, they can be retracted by a conventional catheter.

Soft robotics used for tissue manipulation typically remain inside the body only for the duration of a procedure. Damian et al. have looked toward robotic devices which can allow continual manipulation for an extended duration after implantation [93]. The target purposes of these robotic implants include repairing and improving certain biological processes that react to stimulation in tubular organs; specifically, cell proliferation and tissue growth through applied mechanical forces. To increase the efficiency and safety of the device, sensors can be added for continuous calibration of optimal mechanical force. This would reduce strain on the tissue and prevent possible damage to the cells.

Over the past decade, research into microscale robots have provided prospective engineering solutions for biomedical applications [94]. However, scaling down energy supply components, such as electric motors and batteries, has been a challenge in the development of robotic tools and devices [95]. To combat this, soft microactuators are commonly designed with environmentally responsive materials that experience deformation under environmental changes, such as temperature, light, or pH change. These properties provide potential for untethered actuation. In one example, soft microgrippers made of pNIPAM (a thermoresponsive hydrogel) can achieve self-folding functions by swelling in high temperature water then losing 90% water at low temperatures [92]. The temperature controlled, self-folding actuator also incorporates magnetic nanoparticles, allowing for another level of actuator control by remote direction guidance and gripping of specific tissue at a desired location.

Another application of micro-scale devices is in drug delivery (Figure 4). If soft robots can be actuated by a specific signal, then drugs may be integrated within the device which would be actuated and released at the point of lesion in vivo. This treatment would decrease the side effect for other healthy tissue for efficient drug release. Most microscale soft robotics have the potential to serve as a device for drug delivery because they can respond to specific signals, swim or walk by external guidance, and can be integrated with functional groups such as the micro-gripper [92], or the bioinspired caterpillar [96]. Currently, there are micro devices that directly aim to improve drug delivery and have undergone in vitro testing. The sealed nano straw microdevice which was fabricated by Fox et al. is designed to increase the oral drug absorption under the enzymatic and pH conditions of the gastrointestinal tract. The porous nanostraw membrane controls the drug loading and limits the entry of external molecules [97]. The pH difference between healthy tissue and cancer tissue is used as a signal for micromachines designed by Khezri et al. The graphene oxygen of this device physically absorbs the doxorubicin (DOX), a drug for cancer therapeutic, and releases at a high H+ environment [98]. The advantages of drug delivery by microrobotics are high speed delivery, decreased side effects, and increased efficiency, which has the potential to be applied to in vivo therapeutic applications. Currently, most research regarding soft robotics at the micro-scale shows great potential to non-invasively access human tissue and deliver drugs. However, to date, most experiments are conducted in vitro. Thus, the biocompatibility and efficacy of microscale soft machines and drug delivery systems remain under development.

Lastly, proteins have a complicated structure and can interact with diverse environments. Protein engineering can be used to leverage natural folding processes to build micro and nanoscale soft actuators from biological materials. The development of material structure detection such as X-ray and nuclear magnetic resonance (NMR) contributed to the use of protein-based devices including silk fibroin [99] and fibronectin [9]. Sharma et al. design protein-based motors called viral protein linear (VPL) nano-actuators and demonstrate their function using MATLAB Biokinematics Toolbox as a molecular kinematic computational tool [100]. These protein motors experience conformational changes when a virus attempts to infect a cell and this reaction can be replicated under similar conditions in vitro [101]. Design of protein secondary structure may be used to control function of nanoscale actuators (Figure 4). Prefoldin is a molecular chaperone with a jellyfish-like structure containing six long coiled-coil tentacles and a large central cavity [102]. The long tentacles are flexible and display different conformations in response to changes of pH and temperature [103]. The structure of coils is transformed with the addition of hydrophobic amino acids to capture hydrophobic cargo. The profoldin has shown the ability to interact with many different substrates [104] indicating the probability to achieve more functions combined with varying substrates. Micro- and nanoscale actuators and devices show promise for biomedical applications. As testing of these devices moves from the lab to the clinic, a focus on safety and biocompatibility at the biotic-abiotic interface will prove their strength for translational medical approaches.

## 6. Fabrication Techniques to Enable Micro Soft Robotic Devices

A main challenge in the development of biocompatible micromachines arises from engineering biological materials at the micro and nanoscale and integrating those materials into devices. Recently, multiple methods have been developed and adapted to better suit micromachine biofabrication. These methods include, but are not limited to, 3D printing, nanofiber assembly, colloidal assembly, Janus particle fabrication, and methods of producing films and hydrogels.

### 6.1. 3D Printing

**Figure 5:**
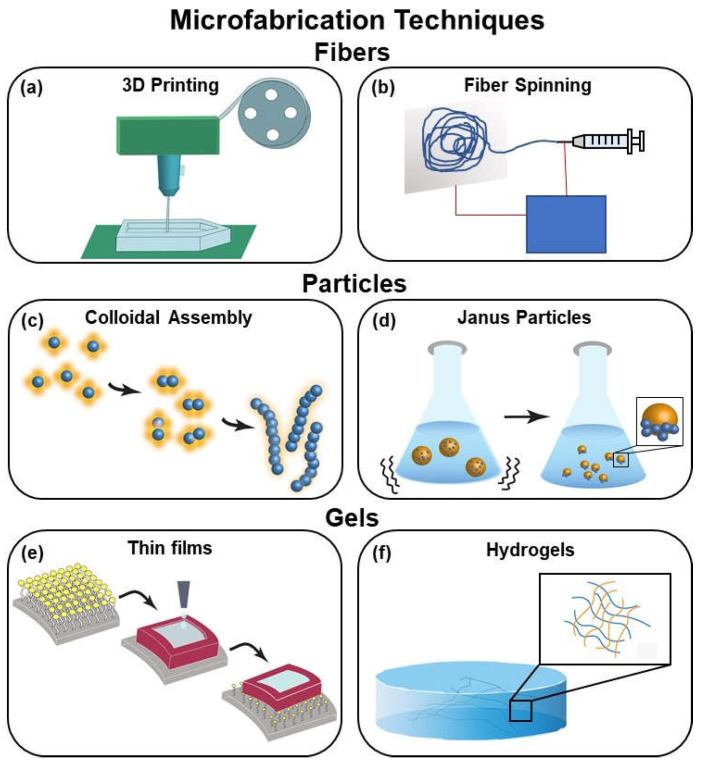
Summary of microfabrication techniques including (**a**) 3D printing, (**b**) fiber spinning, (**c**) colloidal assembly, (**d**) Janus particles, (**e**) thin films, and (**f**) hydrogels.

3D printing is already being employed in the development of medical devices (Figure 1c). With advances in additive manufacturing that increase resolution [105] and incorporate biological materials [106], the application of 3D printing for developing next generation soft devices is clear (Figure 5a). Techniques for 3D bioprinting of soft hydrogels at the micrometer scale are in development and have been successful in printing scaffolds of complex biological structures such as vascular networks and internal organs [107]. One method for benchtop micromachine fabrication which incorporates an SLA 3D printer is the 3D PICL μM (an abbreviation for 3D printing, ink casting, and micromachine lamination) process which is used to produce various biological microdevices. These include microelectrode arrays, microneedles, and microfluidic chips. These devices currently are under development for their potential medical applications in electrophysiology, chip-based disease and organ simulation, drug delivery, gene testing, and environmental monitoring [108].

Hybrid 3D printing is used to prototype multiple microfluidic systems including a finger actuated pump, quick-connect fluidic coupler, and a nucleic acid amplification test device [109]. A “bio-bot” [110], utilized 3D printing and cell culture to achieve motion at the millimeter scale. The bio-bot is displayed as an example of a millimeter scale cell-driven actuator in Figure 4. The hydrogel frame for the bio-bot is stereolithographic 3D printed and embedded with skeletal muscle myoblasts and extracellular matrix (ECM) proteins. The muscle strips were formed through cell culture and then stimulated electrically to cause the muscles to contract. This contraction when confined in the 3D-printed frame, can generate force and enable the bio-bot to crawl at a top speed of about 156 μm/s [110]. 3D printing enables controlled geometry printing of microscale architectures for building next generation soft devices.

### 6.2. Nanofiber Assembly

Pneumatics, a common method for actuation of soft robotic devices, often requires the use of an inextensible layer of fabric. The inextensible layer constrains swelling to facilitate bending via pneumatic actuation. While there are several fabrics capable of forming this inextensible layer at the meter scale, fabricating nanofibers to create this layer at the micrometer and nanometer scale may address the challenges when miniaturizing pneumatic actuators. An early method for nanofiber fabrication is electrospinning, depicted in Figure 5b. Electrospinning can produce polymeric and ceramic nanofibers using electrically charged jets from a high voltage electric field [111]. Electrospinning can be tuned for control over the diameter, shape, structure, and alignment of the nanofibers [112]. The precision of nanofibers assembly has been improved upon significantly to increase the precision and strength of the nanofibers, since the development of the electrospinning method in 1934 [111].

Pull spinning is a rapid nanofiber assembly method which uses a high-speed rotating bristle to form a polymer or protein reservoir into a nanofiber, thus forming a random network of fibers. These fibers can have the composition, orientation, and/or function altered for multiple uses such as textile design, tissue engineering, photonics, and catalysis [113]. While an improvement upon electrospinning, pull spinning is limited to single materials. However, nanofiber composites fabricated with multiple materials have potential applications in regulating various material properties. Rotary jet spinning, a process capable of fabricating nanofibers using multiple materials, builds upon both electrospinning and pull spinning nanofiber assembly [114]. Varying concentrations of the constituent materials, including nylons and polyurethanes, create nanofibers with varying mechanical properties [115]. Nanofiber inextensible layers are formed to assist in the development of soft robotic grippers at the micrometer scale leveraged both MEMs style manufacturing and nanofiber sheets [34]. By varying fiber spinning parameters and geometries, microscale control is possible using these techniques.

### 6.3. Colloidal Assembly

Swarming and assembly are common natural phenomena which enable complex behaviors and functions that cannot be achieved singularly. Swarming and assembly are used to mimic self-organization in nature to produce synthetic micro and nano machines. These machines, commonly referred to as microswimmers, use both fuel-based and fuel-free methods to control colloidal assembly in solution, as illustrated in Figure 5c. Fuels used include enzymes, hydrogen peroxide, and hydrazine. Fuel-free methods include electrical, ultrasonic, light-based, and magnetic manipulation [116]. Colloidal particles, both light active and passive, mimic nature and form complex structures to perform collective functions. This assembly is controlled by instigating light-responsive interactions in the swarm and can be controlled remotely with high precision using optical forces, photochemical reactions, photothermal effects, and photoisomerization. By improving control over this assembly, the goal is to develop adaptable materials and rearrangeable robots for applications such as grasping and transporting at the micro and nano scale [117].

Soft microrobotic artificial muscles, as well as other soft microrobotic devices can be developed using the colloidal nanoparticles as “building blocks”. To assemble the artificial muscle, the colloidal nanoparticles are embedded in stimuli responsive hydrogel nano-actuators which are thermoresponsive and contain magnetized gold [118]. The colloidal assembly is guided by the gold, which forms a nanorod covalently bonded to the hydrogel and nanoparticles. Homogeneous distribution of material for consistent function and performance is almost guaranteed due to the highly ordered colloidal assembly process. Artificial micromuscles are capable of contracting a hydrogel lever arm and arranging into various patterns via magnetic manipulation [118]. Embedding functional particles inside soft materials allows for control of compliant and safe materials for future uses as bioinspired machines.

### 6.4. Janus Particles

The Janus particle is created by combining two materials with different chemical or physical properties at opposite ends to produce a particle with unique and variable functionalities. Janus particles are asymmetrical, as seen in Figure 5d, allowing for self-assembly to enable functions which are not possible for the individual components. Janus particles have potential microfluidic and biomedical applications including sensing, catalysis, imaging, and drug delivery [119]. The particles can be manipulated and guided using various methods including light, magnetism, and pH [120]. Techniques can be used to fabricate active functional surfaces with chemical and topographical control [121]. Using metallic particles, directional control can be achieved using an external magnetic field for applications such as targeted drug delivery (Figure 4) [122]. Magnetic ionic liquid (MIL)-water Janus particles are more difficult to fabricate and are generated with the use of a 3D-printed co-flowing microchannel. Seven flow patterns for the MIL-water Janus particle fabrication are compared to single-phase droplet formation. Control over the arrangement of the surfactant-free MIL-water Janus particle is qualitatively and quantitatively analyzed to demonstrate control using a microdevice and gain further understanding of the controllability of ionic liquid-based Janus particles [123]. These Janus particles can be used for microscale navigation for targeted drug delivery, microscale cargo transport, and assembly as well as nano-jamming structures in soft actuators. The control of these particles can be achieved using bulk guidance with magnetism and heat for control. In the future, they may be used for independent control of micro-swimmers for transport and controlled shape change [124].

### 6.5. Inkjet Printing Films

Polymer materials are often characterized as viscous and flexible, two desirable traits for synthetic soft micromachines. Inkjet printing, a modern but customary approach to fabricating functional materials, can also be applied to viscous elastomers. To test the various material properties that can be achieved using elastomers, the polymer drop coalescence, the process pictured in Figure 5e, was evaluated based on three variables in the printing process. Polymer drop coalescence was most influenced by drop speed but drop spacing and the viscosity of the polymer were not found to influence printing. This insight into inkjet printing will contribute to flexible material fabrication for a variety of micromachines [125]. Electronic circuits which are transparent, flexible, and multilayered have been created using inkjet printing technology. An oil–water interface interaction is used alongside inkjet printing using a silver amine solution in a mosaic silicone structure to wield flexible circuits [126]. Silver nanoparticles can be suspended in PDMS structures. Electronic skins and flexible electrodes are two devices which can be created using this flexible circuit method [127].

Polymer drop deposition also has the potential to be made increasingly biocompatible using biological polymer and protein materials. Microdevices have been fabricated using picoliter-volume inkjet printing and drop-by-drop addition of polymers. The size and morphology of microdevices can be tuned for a multitude of microscale devices with a focus on increasing the efficiency of oral drug delivery [127]. With the continuous development of such fabrication techniques such as 3D printing, nanofiber assembly, particle, and film assembly, highly specific, functional microdevices are within reach. By tuning material chemistry, function, compliance, and biocompatibility can also be tuned and controlled.

## 7. Biocompatibility

**Figure 6:**
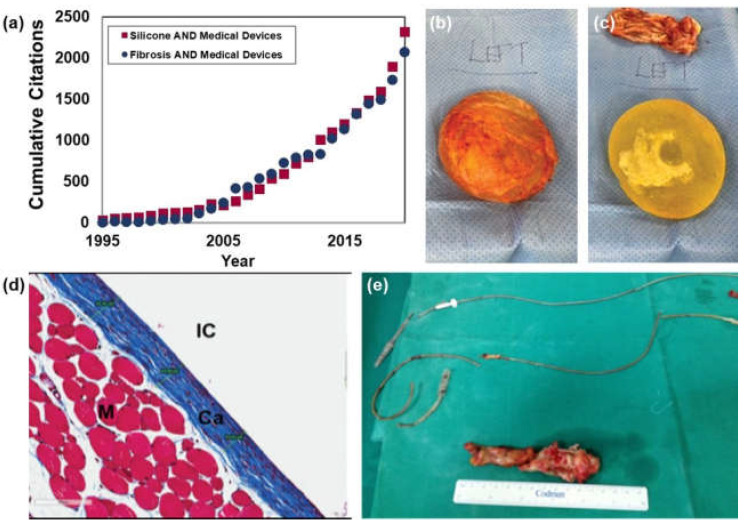
Fibrotic response to implantable devices. (**a**) A Web of Science citation analysis shows that as publications of Silicone Medical Devices were rising in number, so too were citations of work on Fibrosis and Medical Devices. This trend highlights a need to address the fibrosis in the medical device industry. (**b**,**c**) Silicone breast implants are commonly removed due to fibrotic layer development. (**d**) Masson’s trichrome staining on the surface of a silicone implant shows the collagenous layer (Ca, blue) that builds up around the implant (IC) surface. (**e**) Metallic implants such as cardiac pacemaker leads are also subject to the same fibrotic development that inhibits proper function of the device and necessitates removal. (Image (**b**,**c**) adapted from Lee et al. 2020 [130,131], image (**d**) adapted from Frenkiel et al. 2017 [132], image (**e**) adapted from Kokotsakis et al. 2014 [133] http://creativecommons.org/licenses/by/4.0/ (accessed on 1 December 2021)).

While the techniques discussed here hold promise for building microscale devices, to fully integrate soft micromachines into implantable medical devices, considering biocompatibility is critical. Biocompatibility describes a device’s ability to exist within the body with a controlled immune response that does not negatively impact a patient’s health or the overall device performance [128]. When any foreign body is implanted into tissue there is an immune response that follows called the foreign body response (FBR). Throughout the process of wound healing, the body recruits various cells and proteins including leukocytes, neutrophils, fibrin, and growth factors which direct the process of blood clotting and phagocytosis [128]. A fibrotic matrix then begins to form around a foreign body to completely block it from the body. These processes occur to protect the body from perceived danger. When designing implantable devices, however, the inflammatory response that occurs can not only damage the device but also cause rejection, which can be life-threatening [129]. Figure 6a shows an analysis of literature over the past two decades revealing that as the discussion of silicone in medical devices increased, discussions of fibrosis in medical devices followed.

It is critical to control the interactions between implanted materials and soft tissues. The two most basic requirements for a device to be considered biocompatible are the mitigation of cytotoxicity and the ability for the device to withstand biofouling [129]. Variables such as implant location and material properties should be considered when designing a biocompatible device. These include natural, synthetic, or semi-synthetic materials that vary in their structure, porosity, and geometry [128]. Implantation location largely dictates the compatibility of a device as most of the design features listed previously are dependent on biological cues that vary greatly between tissue types [133]. Figure 6 shows examples of elastomer (Figure 6b–d) and metallic materials (Figure 6e) inducing an immune response and fibrotic tissue build up in the breast and heart regions respectively. Material properties such as compliance, longevity, and chemical composition can affect the way tissue interacts with an implanted device [133]. Chemical composition also plays a large role in biocompatibility, as cellular interactions with the device can determine hemocompatibility, the interactions between the device and hematic cells. Factors such as surface charge can affect the body’s immune response, impacting clotting, vascularization, and white blood cell recruitment [134]. If a material is not hemocompatible, an immune response will be triggered that leads to clotting and increased white blood cell counts. Promising research into hemocompatibility, however, has shown that the addition of a thin film of protein on the device alleviates and/or prevents clotting [134]. These responses are particularly dangerous to patients and, if left untreated, can lead to permanent damage to immune systems and the development of autoimmune diseases [133].

There are various forms of in vitro and in vivo assays that can be performed to determine the immune response elicited by these devices [135]. These include using cell cultures to quantify the toxicity of the material by evaluating concentrations of immunoglobulins and other proteins within the immune system [96,136]. Fluctuations in leukocyte concentrations are then used to determine the overall immune response of the body. The extent of degradation of these materials is used to predict the longevity of the device [129]. Table 1 shows a variety of materials, moduli, and the response of these materials in various regions of the body.

Compliance is a property subject to the elasticity of a material. The Young’s modulus of a material can direct numerous mechanical cues to tissues including cell proliferation and differentiation. In general, choosing a material that has a similar compliance or Young’s modulus to that of the surrounding tissue will promote biocompatibility [142,143,147]. A device’s durability will also affect the biocompatibility as a material susceptible to corrosion or untimely degradation will impact device performance as well as potential increases in cytotoxicity. Therefore, most implantable devices use metals such as platinum or stainless steel to avoid corrosion and local changes in chemistry and mechanics [133]. The possibility of corrosion illustrates another motivation to move to corrosion-resistant, soft materials for implantable devices.

A common problem that occurs while designing implantable devices is choosing materials that are both effective and biocompatible. What often happens in these situations is that a certain material is found to be incredibly effective at performing the desired task but induces a toxic inflammatory response within the body. This is often resolved by leveraging hybrid materials or manipulating one of the possible materials to mimic the properties of another [148]. Increasing the biocompatibility of devices opens the door for a variety of bioapplications such as surgical sensors, implantable devices, and targeted drug delivery [149]. Immunosuppressant and anti-rejection drugs that are used post-implantation often have dangerous side effects and can only do so much to prevent the body’s natural immune response [149]. Designing devices that fit a patient’s specific needs and their immune system, will allow for the further integration of soft micromachines into implantable devices.

## 8. Hydrogel-Based Actuators

The use of hydrogels in micromachines is steadily increasing as we learn to manipulate hydrogel properties to fit a variety of applications. Hydrogels, represented in Figure 4 and Figure 5f, at the most basic level, are structured networks of hydrophilic or hydrophobic monomers cross-linked together to absorb water and retain various macromolecules [150]. Hydrogels are especially significant in the design of drug delivery devices and tissue manipulation because of their adaptability in biological environments [42]. Their biocompatibility and tunable mechanical properties make them attractive alternatives to the common soft robot building material, silicone. As a synthetic polymer, its properties are reliable and tunable. That combined with commercial availability make it an appealing biomaterial for implantable devices [151]. Its stiffness, similar to tissue found within the body, make it a popular choice for breast implants and pacemakers [151]. Silicone, however, is known to induce capsular contraction in the tissue surrounding implants. Capsular contraction occurs when the silicone implant induces an FBR and resulting fibrotic capsule [152]. The increased inflammatory response that occurs concurrently with the FBR can not only lead to cell death but also increase the risk of infection [153]. While silicone is used in a variety of applications, it still has the potential to elicit dangerous immune responses post-implantation.

Hydrogels, on the other hand, are most well-known for their biocompatibility. Hydrogels are designed to resemble naturally occurring ECM components commonly found in nature and the body [153]. One of the key features of hydrogels is the fact that they mimic the natural environment to increase biocompatibility. Their tunable mechanical properties such as substrate stiffness, matrix formation, degradation mechanisms, and cellular interactions [154,155] allow them to closely resemble and react similarly to real tissue [142,143]. As a result of their biocompatibility, hydrogels are widely used and act as inspiration for other medical devices [156]. The increased biocompatibility that is often seen with hydrogels drastically decreases the immune system response upon transplantation [157]. As a result, hydrogels maintain a much lower rate of rejection and infection post-transplant when compared to other injectable devices [150].

**Figure 7:**
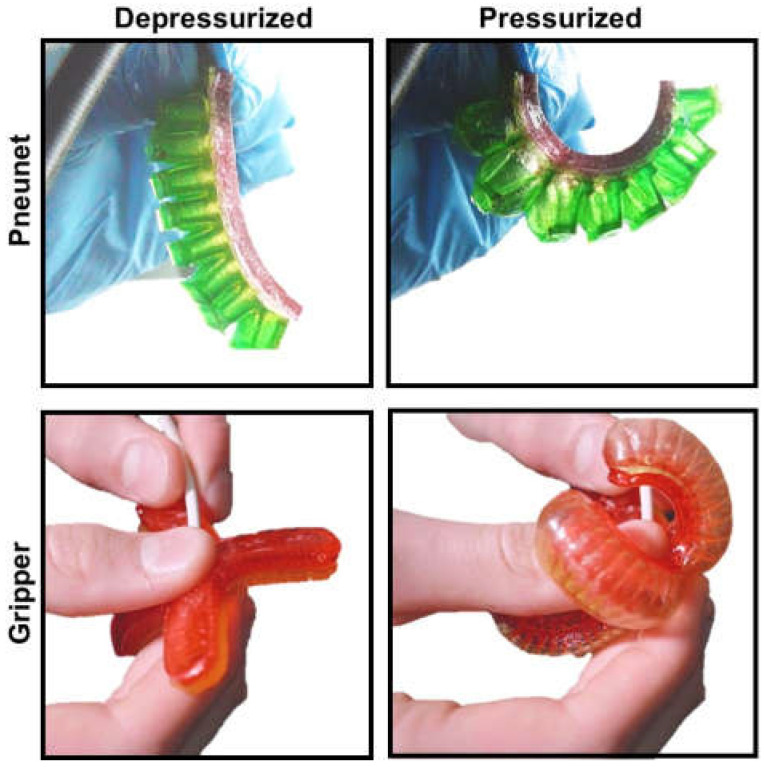
Gelatin based actuators cast as pnuenet fingers and grippers. Actuators can be pressurized with air to induce actuator bending and curling.

The use of hydrogels in implantable devices has proven to be effective at finding a balance between functionality and biocompatibility. This is largely due to the vast variability of materials that can be used to fabricate hydrogels [158]. While there are numerous advantages to using natural materials for the fabrication of hydrogels, they are often difficult to manipulate and produce on a large scale for consistent, implantable devices [159]. Figure 7 shows one example of gelatin-based materials used to build pneunet style actuators first discussed in Section 2.2. The actuators shown here are built according to the protocol in Sardesai et al. [160]. While they are stable in ambient air, this example is not yet water stable. Additional chemistry studies to crosslink these materials will be required to demonstrate functional devices at the millimeter scale. The biocompatibility, vast diversity of materials, and tunable mechanical properties make hydrogels desirable biomaterials for several implantable devices [161]. Engineering these materials into functional robotic devices is the next significant hurdle in the advancement of biocompatible robots. Hydrogels are not only able to effectively mimic biologically relevant environments such as tissue but can be biologically sourced.

## 9. Extracellular Matrix-Based Soft Robotic Actuators

**Figure 8:**
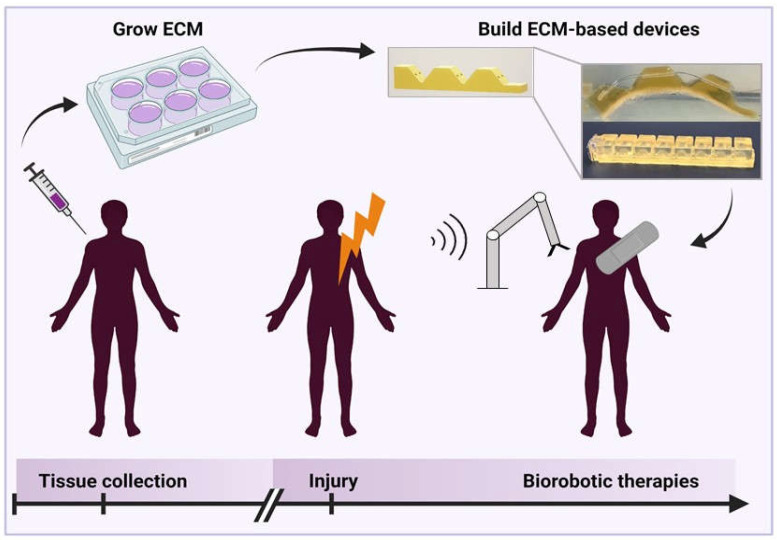
A proposed vision for the future of biorobotic devices in which autologous protein-based (ECM) materials are maintained and engineered into patient specific and disease injury specific devices to be used at the time of injury.

Work is being done to create soft robotic devices out of these biologically sourced hydrogels. It has been shown that soft robots can be made from gelatin [26], collagen, or even gelatin-containing foods (Figure 7) [160]. These robots can mimic the functionality of silicone counterparts [26,150]. It has also been demonstrated that gelatin can be used as a medical device. Currently, in the market there are products like Surgifoam and Gelfoam, sponges that are used to stop bleeding are made from porcine gelatin. However, these are not long-term implantable devices [29,31]. While pure gelatin degrades quickly in aqueous solution, gelatin can be cross-linked using transglutaminase to increase stability and prevent degradation [157], by increasing the bond density within the final gelatin hydrogel, resulting in better force transduction throughout the material. Advances in tissue engineering will enable use of naturally derived or autologous biomaterials as depicted in Figure 8.

In Figure 8 (inset), we show an example of an ECM-based gelatin actuator crosslinked with polycitrate. A hybrid gelatin-polycitrate hydrogel, increases the tensile strength and Young’s modulus [162] of the resulting material. The mechanism behind this is hydrophilic interactions between polar groups on the polycitrate and gelatin molecules, as well as entanglement with the long polycitrate chains [162]. This modification makes the gelatin strong enough to demold without tearing, but more importantly, makes it more stable during testing in aqueous solution, as it can better withstand the internal stresses required for actuation. Using a gelatin-polycitrate hybrid does not impair the biocompatibility of the material as reported in literature [162], as polycitrate is primarily composed of citric acid, and can be hydrolyzed under physiological conditions. In this case, equimolar portions of citric acid and 1,3-propanediol were combined and heated to 140 °C for 25 min while stirring. Simultaneously, a 10% *w*/*v* solution of gelatin in water was mixed at 150 °C until homogenous, then allowed to sit at 150 °C for 15 min until a murky layer formed on top [160]. The solution was then cooled until the top layer was firm enough to remove. The gelatin solution was heated to 150 °C again until liquid, and the polycitrate solution was added at a volume ratio of two parts gelatin to one-part polycitrate and the two were mixed until homogenous. Then the solution was removed from the heat and added to 3D-printed molds to build cable-based and pneumatic actuators. We envision ECM-based devices to represent the future of personalized medical device design.

## 10. Future Outlook

Our analysis of the progression and miniaturization of soft medical devices leads us to surmise what the future of personalized medicine and medical device design might hold. Given the advances in hydrogel and ECM-based structures, combined with fabrication techniques for soft sensors, particles, and fibers, control of microscale soft robotic actuators will increase in resolution as these techniques are tested in combination. As Figure 8 depicts, we envision a future where autologous materials from patients can be cultured over time until they are needed. When patients experience injury or disease, scaffolds, devices, or therapeutics may be built from the stored tissues, on-demand, to meet immediate and personalized medical needs. Clinicians may leverage advances in device design, materials science, and engineering at the biotic-abiotic interface to deliver highly biocompatible, compliant, and personalized medical devices to patients.

## Figures and Tables

**Table 1:** Overview of biocompatibility, modulus, and applications of implantable materials.

Body Region	Material	Modulus	Biocompatible Properties
Skin Grafts	Silk/PLGA hybrid	Silk: 3 MPa–10 GPa [137] PLGA: 7 GPa [138]	These materials are mostly natural polymers or hybrids of natural and synthetic polymers (PLGA), meaning they are biocompatible materials. These materials will not initiate intense immune responses. Large range of Young’s Modulus because of crosslinking, meaning the polymers can replicate the natural environment of the tissue [137,138,139].
Ligaments, Tendons	Silk	Silk: 3 MPa–10 GPa [137]	
	Fibrin	1.7 ± 1.3 MPa (uncrosslinked) [140] 14.5 ± 3.5 MPa (crosslinked) [140]	
Bone Regeneration	Collagen	5–7 MPa [140]	
Heart: Pacemaker Leads Heart Valves, Vascular Grafts	Polyurethane	29–55 GPa [141]	Synthetic polymers have a large range of Young’s Modulus that replicate the various environments in the body. Other synthetic polymers include polylactide, polycaprolactone, and polyglycolide [142,143]. Some synthetic polymers are not as biocompatible as the degradation products of synthetic polymers can be toxic. All materials listed here are FDA approved [139,144,145].
	Polytetrafluoro-ethylene	0.4 GPa [141]	
Eyes	Silicon/Silicone	Silicon: 130–185 GPa [141] Silicone: 3.77 MPa [146]	
Orthopedic Implants (Joints and Support) Dental Implants	Metals (Titanium Alloy and Stainless Steel)	Titanium Alloy: 105–120 GPa [141] Stainless Steel: 180 GPa [141]	These metals are non-corrosive, lending them to be biocompatible in the human body. They are also load-bearing, meaning they can withstand heavy usage, and various movements [133].
